# A Mini Review on the Epidemiology, Transmission, and Clinical Features of Monkeypox Virus

**DOI:** 10.4314/ahs.v26i1.6

**Published:** 2026-03

**Authors:** M Vinoth

**Affiliations:** Department of Chemistry, IFET College of Engineering (Autonomous), Villpuram, Tamil Nadu, 605108, India

**Keywords:** Monkeypox Virus, Orthopoxvirus, Zoonosis, Epidemiology, Antiviral Therapy, Vaccination, Virology

## Abstract

Monkeypox virus (MPXV), a zoonotic member of the Orthopoxvirus genus, has emerged as a significant global health concern following its unprecedented outbreaks beyond endemic regions. The increasing frequency of human-to-human transmission, coupled with viral evolution, necessitates enhanced surveillance and public health interventions. This review provides a critical analysis of MPXV, encompassing its virology, epidemiology, transmission dynamics, clinical manifestations, diagnostic methodologies, therapeutic strategies, and preventive measures. Particular emphasis is placed on the role of vaccination, antiviral therapies, and global response efforts in outbreak mitigation. Furthermore, recent advancements in understanding MPXV pathogenesis, host adaptation, and immune response are explored. Through a critical analysis of recent references, this paper aims to provide an in-depth understanding of monkeypox and propose future research directions to mitigate its spread.

## Introduction

The Monkeypox virus (MPXV) has recently gained widespread global attention due to its potential to cause significant outbreaks, particularly in the wake of the 2022 multi-country event^1^. MPXV is a member of the Orthopoxvirus genus, which includes other clinically significant viruses such as variola virus (responsible for smallpox), vaccinia virus, and cowpox virus^2^. Despite being first identified in captive monkeys in 1958, the primary reservoir of MPXV is believed to be various small mammals, particularly rodents, endemic to Central and West African regions^3^,^4^. Monkeypox was first identified in humans in 1970 in the Democratic Republic of the Congo (DRC), a country thatremains a hotbed for the virus due to recurrent zoonotic transmission and a relatively high burden of disease^5^,^6^. For decades, monkeypox was considered a geographically confined zoonotic infection, primarily affecting individuals in rural and forested regions of Africa who were in close contact with wildlife^7^,^8^. However, increasing globalization, deforestation, and human-wildlife interactions have amplified the virus's capacity to spread beyond its endemic zones^9^,^10^. The cessation of smallpox vaccination programs, which offered cross-protection against MPXV, has further contributed to the increasing vulnerability of populations to MPXV infection^11^. In the years following smallpox eradication in 1980, outbreaks of monkeypox have become more frequent and widespread, indicating that MPXV poses a growing threat to global public health^12^. The 2022 outbreak signified a pivotal shift in the understanding of MPXV epidemiology, with widespread human-to-human transmission observed in regions previously unaffected by the virus, including Europe, North America, and Asia^13^,^14^. While earlier outbreaks were sporadic and primarily linked to zoonotic transmission from infected animals to humans, the recent global outbreak revealed novel transmission patterns^15^. In particular, the high prevalence of infections among men who have sex with men (MSM) and the identification of sexual transmission as a possible mode of spread were critical insights that redefined the epidemiological scope of the virus^16^. The clinical presentation of MPXV infection in humans often mirrors that of smallpox, although typically with less severity, presenting with fever, lymphadenopathy, and a characteristic vesiculopustular rash that can lead to scarring or secondary bacterial infections^17^,^18^. The case fatality rate (CFR) for monkey pox varies by clade, with the Central African (Congo Basin) clade associated with a CFR of up to 10%, while the West African clade, which caused the 2022 outbreak, is associated with a CFR closer to 1-3%. In light of its global resurgence, MPXV has emerged as a pressing topic of concern within the scientific community, necessitating further research into its virology, transmission dynamics, clinical manifestations, and methods for containment^19^,^20^. Although the virus shares several virological characteristics with other orthopoxviruses, such as its large double-stranded DNA genome and complex lifecycle, unique features of MPXV's pathogenesis and immune evasion mechanisms require detailed investigation to inform targeted treatment and prevention strategies^21^. Furthermore, the identification of mutation patterns in the 2022 outbreak strain suggests that the virus may be evolving in ways that enhance its transmission in human populations^22^. Currently, there are no specific antiviral treatments approved solely for MPXV, although several drugs originally developed for smallpox and other orthopoxvirus infections, such as tecovirimat and brincidofovir, have shown promise in treating monkey pox cases. Vaccination, particularly with third-generation smallpox vaccines like JYNNEOS (modified vaccinia Ankara), remains a crucial public health tool for preventing the spread of the virus. However, equitable access to these vaccines, especially in low-resource settings, presents a significant challenge^23^,^24^.

This review aims to provide a comprehensive overview of the current understanding of the monkey pox virus, with a particular focus on recent outbreaks, advancements in diagnostic methods, and the ongoing development of therapeutic and preventive strategies. In doing so, it will synthesize key findings from the most recent literature to offer insights into the evolving epidemiology and virology of MPXV, while highlighting gaps in current knowledge that must be addressed to mitigate the risk of future outbreaks.

## Epidemiology of Monkeypox Virus

### Historical Epidemiology and Geographic Distribution

Historically confined to the tropical rainforests of Central and West Africa, monkey pox cases have surged since the 1970s. The DRC remains the epicenter of endemic transmission, with thousands of suspected cases reported annually. Other affected countries include Nigeria, Cameroon, and the Central African Republic. Between 2017 and 2021, Nigeria experienced a significant resurgence in cases, making it the focus of renewed global attention^25^. The 2022 outbreak marked a shift, with cases reported in North America, Europe, Asia, and the Middle East. Increased global travel, deforestation, and close human-wildlife interactions are among the factors attributed to this spread. Notably, the 2022 strain was linked to the less virulent West African clade, which exhibits a case fatality rate of approximately 1–3%, compared to the 10% rate associated with the Central African clade^26^.

### Recent Outbreaks and Their Public Health Impact

The global 2022 outbreak saw more than 70,000 confirmed cases by late 2022, with the majority of infections occurring outside Africa for the first time. Notably, the transmission pattern during this outbreak differed from previous instances, with a significant number of cases involving men who have sex with men (MSM) and individuals without travel histories to endemic areas^27^,^28^. The outbreak highlighted weaknesses in global health surveillance and preparedness for emerging zoonotic diseases. The various countries with limited historical exposure to MPXV struggled to deploy effective testing and containment strategies, resulting in delays in diagnosis and isolation of cases^29^.

## Virology of Monkeypox Virus

### Genomic Characteristics and Variants

Monkey pox virus is an enveloped, double-stranded DNA virus approximately 197 kilobases in size, encoding over 200 proteins. Its genome structure is similar to that of other Orthopoxviruses, such as variola virus and vaccinia virus, with many of its proteins involved in immune evasion. Two genetic clades of MPXV have been identified: the Central African (Congo Basin) clade and the West African clade, with the latter responsible for the majority of recent global outbreaks^30^. Recent studies have identified genomic mutations in the 2022 outbreak strain, some of which may enhance viral fitness or transmission efficiency^31^. Comparative genomic analysis between the 2022 outbreak strain and earlier strains indicates that the virus has undergone adaptive changes, potentially driven by human-to-human transmission in urban settings^32^.

### Pathogenesis and Immune Evasion

MPXV, like other orthopoxviruses, exhibits various strategies for immune evasion, including the inhibition of interferon responses and the modulation of inflammatory pathways^33^. Studies have demonstrated that MPXV can downregulate major histocompatibility complex (MHC) molecules, thereby evading recognition by cytotoxic T4 lymphocytes. In addition, the virus encodes proteins that inhibit apoptosis, ensuring prolonged survival of infected cells^34^.

## Transmission Dynamics

### Zoonotic Transmission

Monkeypox virus (MPXV) is a zoonotic orthopoxvirus that primarily circulates among wild animal populations, with sporadic transmission to humans. Zoonotic transmission is considered the initial route of human infection, particularly in endemic regions of Central and West Africa. Several studies have identified a variety of potential reservoir hosts, especially small mammals such as rodents. Species including Cricetomys gambianus (Gambian pouched rats), Funisciurus spp. (rope squirrels), and Graphiurus spp. (dormice) have demonstrated serological or molecular evidence of MPXV infection in the wild^35^. These animals are believed to maintain the virus in sylvatic transmission cycles, occasionally leading to spillover events when humans come into contact with them. Human exposure most commonly occurs through direct contact with the bodily fluids, lesions, or respiratory secretions of infected animals, often during hunting, handling, skinning, or preparing bushmeat. The risk is significantly elevated in rural communities where such practices are culturally embedded and economic alternatives are limited. A field investigation by) during an outbreak in the Democratic Republic of the Congo (DRC) revealed that a majority of index cases had recent exposure to wild animals, reinforcing the role of zoonotic transmission as the primary mode of virus introduction into human populations^36^. Furthermore, the 2003 monkeypox outbreak in the United Stateprovided strong evidence for the zoonotic potential of MPXV outside endemic areas. The outbreak was linked to the importation of infected African rodents, which subsequently transmitted the virus to native prairie dogs housed in close proximity. These prairie dogs were sold as pets and became the source of human infections, resulting in 47 confirmed and probable cases^37^. This event demonstrated that non-endemic regions are also vulnerable to zoonotic spillover, particularly through the exotic pet trade. Experimental infection studies, have supported the capacity of various rodent species to harbor and transmit the virus, indicating their potential role as amplifying hosts. Additionally, seroprevalence surveys conducted in endemic zones have consistently identified antibodies against MPXV in wild-caught animals, further validating the existence of natural reservoirs^38^.

### Human-to-Human Transmission

Human-to-human transmission has become increasingly important in recent outbreaks, particularly in urban and non-endemic regions. Transmission between individuals primarily occurs through direct contact with the skin lesions, mucosal surfaces, or bodily fluids of infected persons. Fomites such as contaminated bedding, towels, and clothing also serve as potential vehicles for indirect transmission, especially in shared living environments or healthcare settings. Respiratory droplets may contribute to spread during prolonged face-to-face interactions, although the risk of true airborne transmission appears to be low. The 2022 global outbreak revealed an unexpected epidemiological pattern, with a high incidence of cases among men who have sex with men (MSM), suggesting that close physical and sexual contact may facilitate efficient transmission of the virus in certain social networks^39^. This shift indicates that while monkeypox is not considered a classical sexually transmitted infection, intimate contact can significantly increase the risk of viral spread. The increasing significance of human-to-human transmission underscores the need for robust public health interventions, including rapid diagnosis, isolation of confirmed cases, comprehensive contact tracing, and targeted risk communication. These measures are critical for interrupting transmission chains, particularly in high-density or vulnerable populations. Understanding behavioral and environmental risk factors, as well as potential routes of transmission, remains essential for controlling current outbreaks and preventing future resurgence^40^.

### MSM transmission

Recent monkeypox outbreaks have demonstrated a distinctive epidemiological pattern, with a significant concentration of cases among men who have sex with men (MSM). Unlike previous outbreaks primarily driven by zoonotic exposure in endemic regions, the 2022 multicountry outbreak revealed that sustained human-to-human transmission could occur within dense sexual and social networks, in a cohort of over 500 confirmed cases across 16 countries, 98% were gay or bisexual men, and transmission was strongly associated with recent sexual activity, particularly with multiple partners or anonymous contacts. The clinical presentation in many MSM patients included anogenital lesions and proctitis, which differed from the classical monkeypox rash patterns observed in earlier outbreaks^41^. While monkeypox is not strictly a sexually transmitted infection (STI), the mode of contact close, prolonged skin-to-skin exposure during sexual intercourse facilitates efficient transmission of the virus. In some studies, viral DNA has been detected in seminal fluid, suggesting potential for sexual transmission, although the infectiousness of semen remains under investigation. These findings underscore the need for MSM-specific public health strategies, including risk communication, behavioral guidance, and equitable access to vaccination, without reinforcing stigma. A focused approach to prevention within this group is crucial for containing transmission and maintaining trust between healthcare systems and affected communities^42^.

## Clinical Manifestations

### Incubation Period and Early Symptoms

Monkeypox virus infection begins with an incubation period that can last anywhere from 5 to 21 days, most commonly around 7 to 14 days. During this time, the virus replicates within the host without causing any outward signs or symptoms. After the incubation phase, affected individuals enter the prodromal stage, which is marked by a constellation of non-specific symptoms that often resemble those of other viral infections. These early manifestations include fever, headache, muscle aches, fatigue, and importantly, swelling of the lymph nodes (lymphadenopathy). Lymphadenopathy is a distinctive feature of monkeypox compared to related viruses like smallpox, and it frequently involves nodes in the neck, armpits, or groin. This swelling may appear before the rash, serving as a valuable clinical clue for diagnosis^43^. Following this initial period, a characteristic rash develops, typically within one to three days of fever onset. The rash often starts on the face before spreading to other parts of the body, including the palms and soles. It progresses through several well-defined stages: beginning as flat discolorations (macules), then raised bumps (papules), followed by fluid-filled blisters (vesicles), and eventually pus-filled lesions (pustules). These lesions are usually firm and deeply embedded in the skin, distinguishing them from other rash-causing illnesses^44^. Over a period of two to four weeks, the lesions crust over and fall off, sometimes leaving scars or areas of discoloration. The symptoms during the early phase can be subtle or confused with common infections, which can delay diagnosis and increase the risk of transmission. Moreover, the severity of the clinical presentation can vary depending on factors such as the patient's immune status, age, and presence of coexisting conditions. Children and immunocompromised individuals may experience more severe disease with complications. Understanding the incubation period and early clinical features is essential for healthcare providers to identify cases promptly, implement infection control measures, and reduce the spread within communities^45^.

### Severe Complications and Mortality

Monkeypox virus infection often resolves without severe outcomes in healthy individuals; however, vulnerable groups such as young children, pregnant women, and immunocompromised patients are at greater risk for serious complications. Secondary bacterial infections of skin lesions represent one of the most frequent complications and can lead to cellulitis, abscess formation, or systemic spread left untreated. Respiratory issues, including bronchopneumonia, have also been documented and may significantly worsen the patient's condition^46^. Although uncommon, neurological manifestations such as encephalitis pose substantial risks and have been associated with increased morbidity and mortality. Ocular complications, including conjunctivitis and keratitis, may result in lasting visual impairment without timely management. Several factors influence the occurrence and severity of these complications, including the viral clade, the host's immune response, and the availability of adequate medical care^47^. Differences in mortality rates are notable between the two primary monkeypox virus clades. The Central African (Congo Basin) clade exhibits higher virulence, with fatality rates reported up to 10%, whereas the West African clade generally causes milder diseae, with mortality below 3%. Causes of death frequently involve severe respiratory distress, septicemia, or multi-organ failure. Poor outcomes are often linked to delays in diagnosis and insufficient supportive treatment, highlighting the necessity for rapid identification and proper clinical management. Additionally, factors such as malnutrition and concurrent infections prevalent in endemic areas may exacerbate disease progression. Despite most cases being self-limiting, the potential for serious complications and fatal outcomes underscores the importance of clinical vigilance, strengthened healthcare systems, and preventive strategies including vaccination and public health education to effectively control monkeypox outbreaks^48^.

## Diagnostic Tools

### Laboratory Diagnosis

Reliable laboratory diagnosis is essential for the confirmation of monkeypox virus infection, facilitating timely clinical management and effective public health interventions. Among available methods, polymerase chain reaction (PCR) is regarded as the most sensitive and specific technique for detecting monkeypox viral DNA. This molecular assay is typically performed on samples collected from skin lesions, including swabs of vesicular or pustular fluid, crusts, and sometimes respiratory secretions. PCR assays target highly conserved regions of the orthopoxvirus genome, enabling not only detection but also differentiation between monkeypox and other orthopoxviruses such as variola (smallpox) and vaccinia viruses. Although PCR offers rapid and definitive results, its requirement for specialized laboratory infrastructure and trained personnel limits its accessibility in resource-constrained settings^49^. Serological assays, such as enzyme-linked immunosorbent assay (ELISA) and immunofluorescence assays, can identify antibodies produced against orthopoxviruses, providing useful information for retrospective diagnosis and epidemiological studies^50^. However, cross-reactivity with antibodies from previous smallpox vaccination or exposure to other orthopoxviruses complicates interpretation, and these tests have limited utility in acute infection diagnosis due to the delay in antibody production. Virus isolation remains the definitive method for confirming infection but requires biosafety level 3 (BSL-3) or higher laboratories and is time-consuming, which restricts its routine application during outbreaks. Recent advances focus on developing rapid point-of-care diagnostic tests and isothermal amplification techniques, such as loop-mediated isothermal amplification (LAMP), to overcome logistical challenges and improve surveillance in endemic and outbreak settings. The integration of such innovative diagnostic tools, alongside PCR and serology, can enhance early case detection, inform patient care, and support containment efforts during monkeypox outbreaks^51^.

### Point-of-Care Testing and Innovations

The emergence of point-of-care (POC) diagnostic technologies has revolutionized the approach to detecting monkeypox virus, especially in settings where access to centralized laboratory facilities is limited or delayed. These innovations focus on delivering rapid, accurate, and easily accessible diagnostic solutions directly at the site of patient care, which is critical for prompt isolation, treatment initiation, and containment of outbreaks. Among the most promising advancements are isothermal nucleic acid amplification techniques such as loop-mediated isothermal amplification (LAMP) and recombinase polymerase amplification (RPA). Unlike traditional polymerase chain reaction (PCR) assays, these methods do not require thermal cycling equipment, allowing amplification of viral genetic material at a constant temperature^52^. This feature reduces both the complexity and cost of testing, making them suitable for use in resource-constrained environments. In addition to nucleic acid amplification, lateral flow immunoassays (LFIA) have been developed to detect monkeypox virus antigens or host antibodies rapidly. These paper-based tests produce results within minutes and require minimal technical expertise, which facilitates their use in field investigations and during rapid response efforts. Recent designs incorporate portable, battery-operated devices and smartphone-based readers that not only improve the accuracy and interpretation of results but also enable real-time data reporting to public health authorities, thus enhancing epidemiological surveillance. Despite these promising developments, challenges remain in ensuring the sensitivity, specificity, and overall reliability of POC tests^53^. Comprehensive validation studies are essential before widespread adoption to prevent false negatives or positives that could hamper outbreak control efforts. Continued investment in research and development of POC diagnostics is vital to strengthening global preparedness and response capabilities against monkeypox and other emerging infectious diseases^54^.

## Treatment and Management

### Antiviral Therapies

Although no antiviral medication has been specifically approved for monkeypox virus infection to date, several drugs developed for related orthopoxviruses are being evaluated for their therapeutic potential. Tecovirimat (also known as TPOXX or ST-246) is the most prominent among these agents. It functions by inhibiting the viral envelope protein VP37, which is essential for the production and dissemination of extracellular virus particles. Preclinical studies using animal models have demonstrated that tecovirimat can significantly reduce viral replication and improve survival rates following orthopoxvirus infection^55^. Compassionate use cases in humans have also shown promising results, suggesting that this antiviral may reduce disease severity and duration. Additionally, cidofovir, a nucleotide analogue that inhibits viral DNA polymerase, has demonstrated in vitro activity against monkeypox and other orthopoxviruses; however, its clinical use is limited by potential nephrotoxicity and intravenous administration. Brincidofovir, an oral lipid conjugate of cidofovir, offers improved bioavailability and a better safety profile, though clinical evidence for its effectiveness against monkeypox remains scarce. Supportive care remains fundamental in managing monkeypox patients, emphasizing symptom relief, prevention of secondary bacterial infections, maintenance of fluid and electrolyte balance, and nutritional support^56^. Given the heterogeneous clinical presentations and potential for severe complications, individualized treatment plans are essential. As monkeypox cases rise globally, especially in regions outside traditional endemic areas, the need for well-designed clinical trials to assess the safety and efficacy of these antiviral agents becomes increasingly urgent. Furthermore, integrating antiviral therapies with vaccination strategies and robust public health interventions will be critical to controlling outbreaks and improving patient outcomes. Continued research into novel antiviral compounds and treatment regimens is necessary to expand the therapeutic arsenal against this re-emerging viral threat^57^.

### Supportive Care and Complications

Supportive care is the primary approach to managing monkeypox virus infection, given the lack of specific antiviral treatments widely available for routine use. This care centers on symptom management, maintaining patient comfort, and preventing secondary complications that can significantly worsen outcomes. Symptomatic treatment often includes the use of antipyretics and analgesics to reduce fever and alleviate pain caused by the characteristic vesicular-pustular rash and associated lymphadenopathy^58^. Pruritus control is also important to prevent excessive scratching, which can lead to secondary bacterial infections. Adequate hydration and nutritional support are vital components of care, particularly in patients with extensive mucocutaneous involvement who may experience difficulties in oral intake due to painful lesions in the mouth and throat. Secondary bacterial infections remain a common and serious complication in monkeypox patients, often requiring prompt antibiotic therapy to prevent progression to more severe conditions such as cellulitis or sepsis. Beyond cutaneous complications, systemic manifestations can include bronchopneumonia, which arises from viral or bacterial infection of the lungs, and has been associated with increased morbidity. Neurological complications such as encephalitis, although rare, pose significant risks and necessitate vigilant monitoring and early intervention^59^. Ocular involvement, including conjunctivitis and keratitis, may lead to vision impairment if not addressed promptly. These complications disproportionately affect vulnerable groups, including children, pregnant women, and immunocompromised individuals, who may experience more severe disease courses and higher mortality rates. Furthermore, supportive care strategies must be integrated with public health measures to control outbreaks and minimize transmission. Ongoing research into the pathophysiology of complications and the development of targeted therapies will be critical to enhancing clinical management and patient outcomes in future monkeypox outbreaks^60^.

## Prevention and Vaccination

### Vaccination Strategies

Vaccination continues to be a pivotal component in the prevention and control of monkeypox virus infection, especially as the virus re-emerges beyond its traditional endemic regions. Historically, the smallpox vaccine, which utilizes a live vaccinia virus, has demonstrated significant cross-protection against monkeypox due to the close genetic relationship between the two orthopoxviruses^60^. This cross-immunity was a major factor contributing to the decline in monkeypox cases following the global smallpox eradication campaign. However, the cessation of routine smallpox vaccination decades ago has resulted in waning immunity in the general population, increasing susceptibility to monkeypox infections^61^. To address this gap, newer-generation vaccines, such as the modified vaccinia Ankara (MVA) vaccine, have been developed. MVA is a highly attenuated, non-replicating vaccine designed to offer enhanced safety, particularly for individuals who are immunocompromised or have contraindications to traditional replicating vaccinia vaccines. Vaccination strategies against monkeypox typically include both pre-exposure and post-exposure approaches. Pre-exposure vaccination is prioritized for individuals at high risk, such as healthcare workers, laboratory personnel handling orthopoxviruses, and people in endemic or outbreak-affected areas^62^. Post-exposure prophylaxis, ieally administered within four days of exposure, can reduce the risk of disease onset and severity. One of the most effective public health measures during outbreaks has been the implementation of ring vaccination, which involves vaccinating close contacts of confirmed cases and their contacts to create a buffer that interrupts viral transmission chains. This strategy proved instrumental during previous monkeypox outbreaks and remains a cornerstone of outbreak response. Expanding vaccine availability and coverage, especially in non-endemic countries currently facing emergent cases, is critical to preventing further spread. Challenges such as vaccine hesitancy, logistical distribution barriers, and limited stockpiles must be addressed through coordinated public health efforts. Furthermore, ongoing research into the duration of vaccine-induced immunity, the effectiveness of different vaccine platforms, and potential booster requirements is essential to inform long-term vaccination policies. When combined with surveillance, contact tracing, and community education, vaccination forms a comprehensive framework for controlling monkeypox virus transmission and protecting vulnerable populations worldwide^63^.

### Public Health Interventions

Robust public health interventions are fundamental to curbing the transmission of monkeypox virus and mitigating its impact on affected populations. Central to these efforts is the establishment of comprehensive surveillance systems that enable early detection and rapid reporting of suspected cases. Enhanced surveillance facilitates timely isolation of infected individuals, thereby interrupting transmission chains^64^. Contact tracing plays a pivotal role by identifying individuals who have been exposed to confirmed cases, allowing for close monitoring and prompt implementation of quarantine measures when necessary. Public health authorities must also prioritize clear and culturally sensitive communication strategies to educate communities about the virus, its symptoms, and effective prevention practices^65^. Such educational initiatives are crucial in dispelling misinformation, reducing stigma associated with the disease, and encouraging affected individuals to seek medical attention early. Healthcare settings, stringent infection prevention and control (IPC) protocols are critical to safeguarding healthcare workers and other patients^66^. This includes the proper use of personal protective equipment (PPE), safe handling and disposal of contaminated materials, and rigorous environmental cleaning^67^. Training healthcare personnel in IPC measures and outbreak response enhances the overall resilience of health systems during monkeypox outbreaks^68^. Community engagement and collaboration with local leaders and organizations help to tailor interventions to the social and cultural context, increasing public trust and compliance with health directives. During outbreaks, public health responses often incorporate vaccination campaigns, including ring vaccination strategies that target contacts of confirmed cases and their immediate networks. Travel advisories and screening at points of entry can also limit the geographical spread of the virus^69^,^70^. Furthermore, public health policies must be adaptable, relying on real-time data and epidemiological insights to refine intervention strategies as the outbreak evolves. Intersectoral collaboration among government agencies, international organizations, and research institutions is essential to mobilize resources, share knowledge, and coordinate actions effectively. Together, these multi-faceted public health interventions form a critical defense against monkeypox virus transmission and contribute to safeguarding global health security^71^.

## Conclusion and Future Perspectives

The resurgence of monkey pox as a global health concern underscores the need for continued research into its virology, transmission dynamics, and effective prevention and treatment strategies. While the 2022 outbreak has largely been contained, the potential for future outbreaks remains high, particularly in regions with poor healthcare infrastructure or where human-animal interactions are frequent. Future research should focus on the development of targeted antiviral therapies, rapid diagnostic tools, and vaccine strategies to mitigate the impact of MPXV on global health. Collaborative efforts between governments, public health agencies, and researchers will be essential in addressing these challenges.
